# Supplementary material for: Biology System Description Language (BiSDL): a modeling language for the design of multicellular synthetic biological systems
Source: BMC Bioinformatics. 2024 Apr 25;25:166. doi: 10.1186/s12859-024-05782-x (PMC11046772; doi:10.1186/s12859-024-05782-x)
Supplement: Supplementary file 1 — Additional file 1: BiSDL Modules Library. Appendix illustrating the syntax and semantics of the basic building blocks used to create a Biology System Description Language (BiSDL) description (Section 1) and the mapping between BiSDL constructs and the chosen NWN representation (Section 2). [file 12859_2024_5782_MOESM1_ESM.pdf]

Additional file 1

- Format: `.pdf`
- Title: BiSDL Modules Library
- Description: Appendix file illustrating the syntax and semantics of the basic building blocks used to create a Biology System Description Language (BiSDL) description (Section 1) and the mapping between BiSDL constructs and the chosen `nwn-snakes` representation (Section 2).

BiSDL Modules Library

1 Basic building blocks

This work proposes a library of BiSDL constructs as an exemplification of its semantic capabilities that show the expressiveness and closeness to the biological semantics of the language. This section illustrates the syntax and semantics of the basic building blocks used to create a BiSDL description, focusing on accessibility and biological significance. Since BiSDL aims to be an easily readable language, it uses English keywords and minimal punctuation. It also avoids cumbersome solutions to delimit blocks. To this end, the use of indentations is suggested, although not mandatory, to set each block apart from the surrounding code visually.

The provided building blocks belong to different domains at Level II, where they provide descriptions of basic biological concepts, including simple local relations in the spatial domain, such as the relative position of two biological elements, and base functions in the behavioral domain, such as the production or degradation of a protein.

1.1 Process building blocks

This section provides a detailed overview of the basic constructs offered by BiSDL to model more complex behaviors inside `PROCESS` constructs. All parameters accepted by these building blocks are the base BiSDL types `gene`, `mrna`, `protein`, (protein) `complex`, and generic `molecule`, collectively indicated as *molecules* in the following. All molecule parameters can optionally be associated with a multiplier, i.e., an integer number indicating the ratio between the molecules involved in the process.

Listing 1: Transcription building block template

```
TRANSCRIPTION(<gene>, <mrna> (, regulation))
```

The construct in Listing 1 models the copying of DNA segments that can encode proteins in mRNA, the first step in gene expression, with the following parameters:

- `<gene>`: the gene to be transcribed; must end in "_gene";
- `<mrna>`: the transcribed mRNA; must end in "_mrna";
- (optional) `regulation` is a list of molecules acting as inhibitors, inducers, or activators (see subsection 1.2).

Listing 2: Transcription building block example

```
TRANSCRIPTION(GFP_gene, GFP_mrna)
```

Listing 2 provides an example of the transcription of a gene to an mRNA.

Listing 3: Translation building block template

```
TRANSLATION(<mrna>, <protein> (, regulation))
```

The construct in Listing 3 models the process of protein synthesis from their mRNA blueprint, with the following parameters:

- `<mrna>`: the mRNA to be translated; must end in "_mrna";
- `<protein>`: the protein to be produced; must end in "_protein";
- (optional) `regulation` is a list of molecules acting as inhibitors, inducers, or activators (see subsection 1.2).

Listing 4: Translation building block example

```
TRANSLATION(GFP_mrna, 2*GFP_protein)
```

Listing 4 provides an example of the translation of a mRNA to two proteins.

Listing 5: Degradation building block template

```
DEGRADATION(<molecule>)
```

The construct in Listing 5 models degradation of a molecule, with the following parameter:

- `<molecule>`: the molecule to be broken down.
- (optional) `regulation` is a list of molecules acting as inhibitors, inducers, or activators (see subsection 1.2).

Listing 6: Degradation building block example

```
DEGRADATION(3*GFP_protein)
```

Listing 6 provides an example of the degradation of a protein.

Listing 7: Complex formation building block template

```
PROTEIN_COMPLEX_FORMATION(<molecule>, <molecule>, ...,
    <molecule>)
```

The construct in Listing 7 models the process of two or more proteins associating in a protein complex, with the following parameter:

- `<molecule>, ...`: the proteins (at least two) that participate in the formation of the complex, followed by the name of the protein complex itself.

Listing 8: Complex formation building block example

```
PROTEIN_COMPLEX_FORMATION(3OC6HSL_molecule, LuxR_protein,
    3OC6HSL_LuxR_complex)
```

Listing 8 provides an example of the formation of a protein complex.

Listing 9: Enzymatic reaction building block template

```
ENZYMATIC_REACTION(<protein>, [<molecule>, ...], [<molecule>,
    ...])
```

The construct in Listing 9 models the enzyme catalysis mechanism, through which a catalyst facilitates the reactants in the formation of one or more products, with the parameters:

- `<protein>`: the catalytic enzyme;
- `[<molecule>, ...]`: a list of at least one reactant, i.e., the *input* molecules;
- `[<molecule>, ...]`: the list of at least one product formed in the process, i.e., the output molecules.

Listing 10: Enzymatic reaction building block example

```
ENZYMATIC_REACTION(Lux1_protein, [SAM_molecule, ACP_molecule],
    [_3OC6HSL_molecule])
```

Listing 10 provides an example of an enzymatic reaction with two reagents and one product.

Listing 11: Custom process building block template

```
CUSTOM_PROCESS([<molecule>, ...], [<molecule>, ...] (,
    regulation))
```

The construct in Listing 11 allows the modeling of biological concepts not covered by the previously described ones, with the parameters:

- `[<molecule>, ...]`: a list of at least one reactant, i.e., the *input* molecules;
- `[<molecule>, ...]`: the list of at least one product formed in the process, i.e., the *output* molecules;
- (optional) `regulation`: a list of molecules acting as inhibitors, inducers, or activators (see subsection 1.2).

Listing 12: Custom process building block example

```
CUSTOM_PROCESS([2*H2_molecule, O2_molecule], [2*H2O_molecule])
```

Listing 12 provides an example of a custom process.

1.2 Regulation

It is possible to optionally specify a list of regulatory mediators for the TRANSCRIPTION, TRANSLATION, DEGRADATION, and CUSTOM_PROCESS constructs, to increase or decrease the yield of the process.

Listing 13: Regulation mechanism template

```
<regulation\_type>: <molecule>, ... (, <regulation\_type>: ...)
```

Listing 13 shows the regulation construct, that accepts the following parameters:

- `<regulation_type>`: `INDUCERS`, `INHIBITORS`, or `ACTIVATORS`;
- `<molecule>, ...`: a list of molecules acting as inhibitors, inducers, or activators.

Listing 14: Regulation mechanism example

```
TRANSCRIPTION(GFP_gene, GFP_mrna, INDUCERS: 3OC6HSL_LuxR_complex)
```

Listing 14 exemplifies the transcription of a gene to an mRNA, induced by a protein complex.

1.3 Signaling mechanisms

The signaling mechanisms available in BiSDL enable the exchange of specific information between SCOPE entities:

- from a source entity towards its neighbors;
- from a source entity to a linked destination entity;
- bidirectionally, between two entities.

The first two – implemented by the constructs PARACRINE_SIGNAL and JUXTACRINE_SIGNAL, respectively – must be specified inside the SCOPE context, as they rely on the properties of the enclosing biological compartment. On the other hand, the specification of the third one (i.e., the DIFFUSION construct) must be inside the MODULE context after the description of all SCOPE statements, as it is a property equally shared between two SCOPE statements.

The BiSDL types that can be involved as signal parameters are the protein, complex, and molecule types (i.e., gene, mrna, and receptor types are not signals).

Listing 15: Paracrine signaling template

```
PARACRINE_SIGNAL(<signal>, ...)
```

Listing 15 models the communication mechanism from a cell to its neighboring cells. It requires the specification of a signal (or a list of signals). Spatial coordinates allow for automatically inferring the neighborhood of the container SCOPE. The signals reach the destination SCOPE unchanged. It accepts the following parameters:

- <signal>, ...: one or more protein or molecule entities acting as a signal;

Listing 16: Juxtacrine signaling template

```
JUXTACRINE_SIGNAL(<signal> -> <entity_name>)
```

Listing 16 models contact-dependent signaling from a cell to a specific linked cell. It requires the signal and the receiving entity to be specified, with the following parameters:

- <signal>: the protein or molecule entity acting as a signal;
- <entity_name>: the destination SCOPE.

If the signal is a molecule (i.e., its name contains the tag "_molecule"), the corresponding construct models a cellular junction through which the signal is transferred from the source compartment to the destination one (Figure 1). On the other hand, if the signal involved is a protein (i.e., its name contains the tag "_protein"), it is interpreted as a membrane ligand and *consumed* in the bond with an active receptor (Figure 2 and Figure 11).

As a first example, the BiSDL module in Listing 17 models the mock process depicted in Figure 1 and is compiled to the Petri Nets (PNs) in Figure 10, where a communicating junction links two adjacent cells, allowing the molecule's transit, where it is modeled with the Nets-Within-Nets (NWNs) formalism as follows.

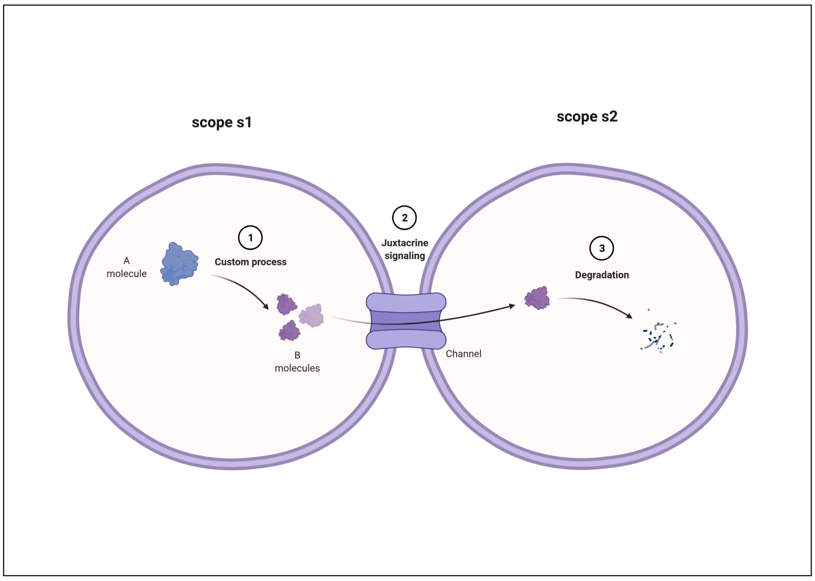

Fig. 1: This mock process modeled in Listing 17 involves two biological scopes, `s1` and `s2`. `s1` is the location where three `B_molecules` are produced (1) starting from one `B_molecule`. `B_molecules` are allowed to transit through a communicating junction from scope s1 to scope s2 (2). In `s2`, `B_molecules` are constantly degraded (3).

Listing 17: Modeling junction-mediated juxtacrine signaling

```
MODULE example
    TIMESCALE 1
    SCOPE s1 (0, 0)
        PROCESS p1
            TIMESCALE 1
            CUSTOM_PROCESS ([A_molecule], [3*B_molecule])
        JUXTACRINE_SIGNAL B_molecule -> s2
    SCOPE s2 (0, 1)
        PROCESS p2
            TIMESCALE 1
            DEGRADATION (B_molecule)
```

As a second example of juxtacrine signaling, Listing 18 models the biological system in Figure 2, where two adjacent cells interact through a membrane ligand (`A_protein`) and a membrane receptor (`A_receptor_active_protein`).

Listing 18: Modeling ligand-receptor juxtacrine signaling

```
1  MODULE example
2      TIMESCALE 1
3      SCOPE cell_A (0, 0)
4          PROCESS process_A
5              TIMESCALE 1
6              TRANSCRIPTION (A_gene, A_mrna)
7              TRANSLATION (A_mrna, 3*A_protein)
8              DEGRADATION (A_protein)
9          JUXTACRINE_SIGNAL A_protein -> cell_B
10     SCOPE cell_B (0, 1)
11         PROCESS process_B
12             TIMESCALE 1
13             DEGRADATION (A_receptor_active_protein)
```

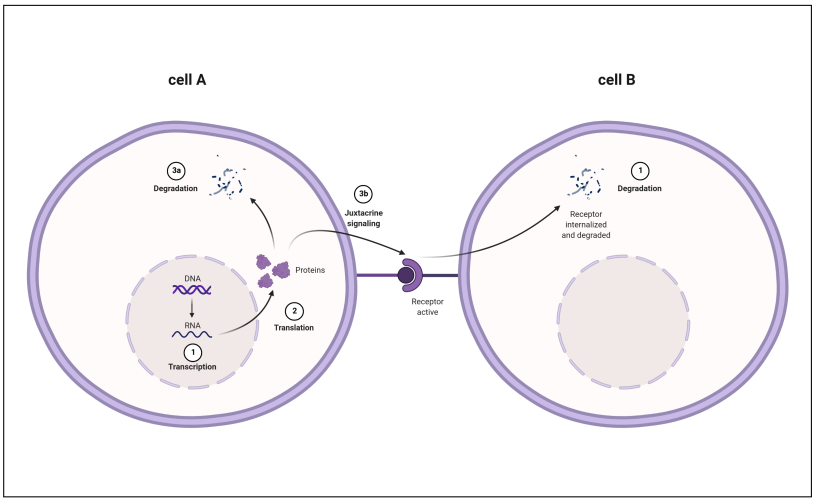

Fig. 2: This mock process modeled in Listing 18 involves two biological scopes, `cell_A` and `cell_B`. `cell_A` is the location where three `A_molecules` are produced (2) starting from gene-mRNA transcription (1). `A_molecules` may face degradation (3a), or they could bind (3b) the corresponding receptor on `cell_B` membrane. The active receptor is subject to degradation (`cell_B`, (4)).

In BiSDL, the declaration of the juxtacrine signaling from `cell_A` to `cell_B` (line 9, Listing 18) using the `A_protein` ligand builds the whole ligand-receptor underlying structure. That is, `SCOPE cell_B` is automatically endowed with the (virtual) permanent presence of a membrane receptor for `A_protein` ligand. When the `juxtacrine_signaling_A_molecule_s1_s2_1` transition (Figure 11.a) fires, one `A_protein` token is consumed in `cell_A` and one `A_receptor_active_protein` token is produced in `cell_B`, with the text substitution signifying the activation of the bond. Therefore, there is no need for additional modeling of the signaling in the description

of SCOPE cell_B behavior (lines 10-13, Listing 18). The DEGRADATION process (line 13, Listing 18) models the degradation of ligand-receptor complexes.

Listing 19: Diffusion template

```
DIFFUSION  <entity_name>, <entity_name>, <signal> (, <signal>,
    ...)
```

Listing 19 models a bi-directional permeable membrane between two biological districts, allowing the movement of signals between them, with the following parameters:

- `<entity_name>, <entity_name>`: the two communicating SCOPE entities;
- `<signal>, ...`: one or more signal entities the virtual membrane is permeable to.

Listing 20: Modeling diffusion

```
1  MODULE example
2      TIMESCALE 1
3      SCOPE s (0, 0)
4          PROCESS ps
5              TIMESCALE 1
6              CUSTOM_PROCESS ([A_molecule, B_molecule],
                   [C_molecule])
7      SCOPE t (0, 1)
8          PROCESS pt
9              TIMESCALE 1
10             DEGRADATION (A_molecule)
11             DEGRADATION (C_molecule)
12     DIFFUSION s, t, [A_molecule, 2*C_molecule]
```

Listing 20 is the BiSDL representation of the biological phenomena in Figure 3. A biological district is divided in two by a membrane. In the leftmost part (a) in Figure 3, a reaction occurs through which C_molecule is produced starting from A_molecule and B_molecule. The membrane is permeable to molecules A and C, with a higher permeability for C. This different permeability is modeled in BiSDL using a multiplier (line 12, Alg. 20).

2 From BiSDL to NWNs

This section details the mapping between BiSDL constructs and the chosen NWNs target representation through the nwn-snakes library. The Module class is a utility layer that eases the simulation task, automatically wrapping the NWNs model structure compiled from the BiSDL model description. To facilitate the declaration of variables, the specification of NWNs elements in the Module class follows the order: PNs, places, Transitions, and finally input and output arcs involving the preceding elements of any level. Each BiSDL PROCESS (see Section 1.1), once compiled, maps to a net token where places and transitions deal only with black tokens. The black tokens at the lower level correspond, in the top-level *container* place, to colored tokens, i.e., strings bearing the name of the lower-level place holding them. places that are shared

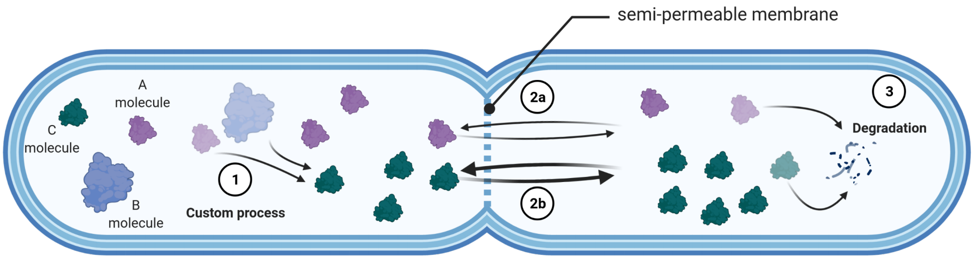

Fig. 3: This mock process modeled in Listing 20 involves two biological compartments separated by a semi-permeable membrane allowing the diffusion of `A_molecule` and `C_molecule`. The latter diffuses (2b) twice *as easily* as the former (2a) through the membrane. The compartment left to the membrane is the location where `C_molecule` is produced (1) starting from one `A_molecule` and one `B_molecule`. In the rightmost compartment, the only transformation is the degradation (3) of molecules A and B.

among constructs of the same `PROCESS` context are instantiated only once, while their interactions (in terms of arcs and transitions) are created accordingly to the modeled processes.

2.1 Compiled process building blocks

As an example, The BiSDL `TRANSLATION` construct in Listing 4 compiles into the PNs shown in Figure 4, having one `GFP_mrna` place and one `GFP_protein` place. The two are connected by one `GFP_mrna_translation` transition that, once fired, consumes one token from the `GFP_mrna` place and produces two in the `GFP_protein` place. In this example process, where two proteins (the `(dot, dot)` black tokens) are produced when one mRNA black token (`dot`) is consumed.

As another example, the BiSDL `DEGRADATION` construct in Listing 6 compiles into the PNs shown in Figure 5: a `GFP_protein` place is created and connected to three separate transitions that can fire independently, thus depleting up to three tokens.

For the `ENZYMATIC_REACTION` construct, Listing 10 compiles into the PNs shown in Figure 6. In this example, `Lux1_protein` catalyzes the reaction process involving the reactants `SAM_molecule` and `ACP_molecule`, leading to the formation of `3OC6HSL_molecule`. A place is created for the catalytic enzyme and one for each reactant and product. The reactants are input places for the transition representing the enzymatic reaction, which produces one token in the `3OC6HSL_molecule` place upon firing. At the same time, one token is produced back in the `Lux1_protein` place, which, being a catalyst, is not consumed nor changed by the reaction.

The BiSDL `CUSTOM_PROCESS` construct in Listing 12 compiles into the PNs shown in Figure 7: `H2_molecule` and `O2_molecule` participate in the formation of `H2O_molecule`. One place is created for each molecule. When the transition fires, two black tokens are produced in the `H2O_molecule` place, while two black tokens are depleted from the `H2_molecule` and one from the `O2_molecule`.

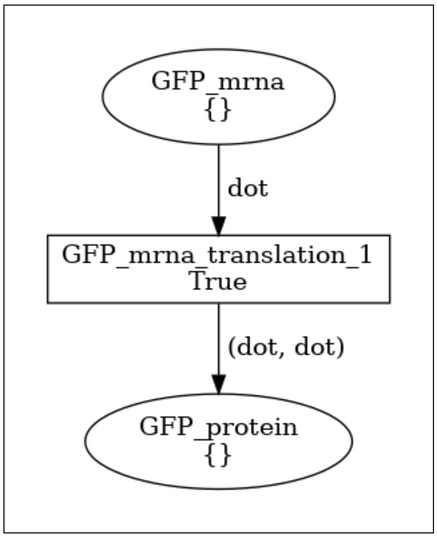

Fig. 4: PNs for the translation process, generated from the BiSDL code for `TRANSLATION` in Listing 4. Two proteins (the `dot, dot` black tokens) are produced when one mRNA black token is consumed.

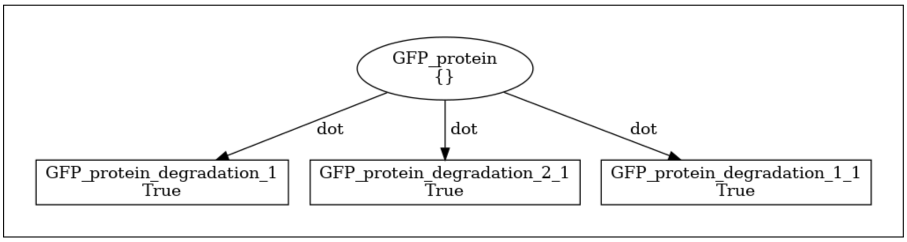

Fig. 5: PNs for the degradation process corresponding to the construct for `DEGRADATION` in Listing 6. When each one of the transitions fires, one protein is consumed.

In general, BiSDL descriptions holding these constructs generate a two-level hierarchy of nets. For example, in the context of a complete BiSDL description, the `CUSTOM_PROCESS` construct in Listing 12 generates the described PNs model (see Figure 7, and 8.b) at the lower level, and a single place holding it at the top level (Figure 8.a).

2.2 Compiled regulation constructs

To obtain regulation of basic building block operation, inducer molecules are mapped to additional input places to their main transition. As an example, The BiSDL `TRANSCRIPTION` construct in Listing 14 compiles into the PNs shown in Figure 9, including a `GFP_gene` place, a `GFP_mrna` place, and a `3OC6HSL_LuxR_complex` place,

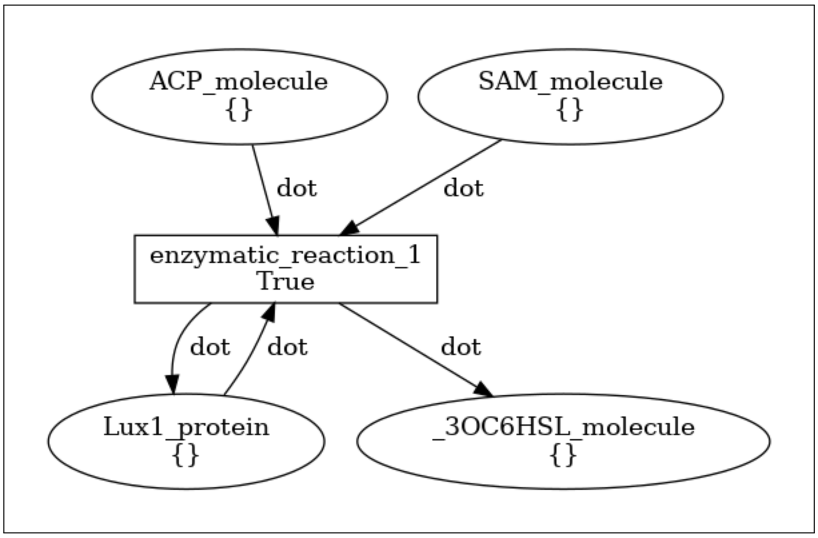

Fig. 6: PNs for the enzymatic reaction process corresponding to the construct for `ENZYMATIC REACTION` in Listing 10. When the transition fires, one black token is consumed from each of the input places, and one token is produced in the output place (`3OC6HSL_molecule`) and in the place representing the catalysts (`Lux1_protein`).

connected by one transition. The `GFP_gene_transcription` transition has two input arcs from the `GFP_gene` place and the `3OC6HSL_LuxR_complex` place and two output arcs towards the `GFP_gene` and the `GFP_mrna` place. The gene place is automatically initialized with one black token. The gene token is consumed when the transition fires and is immediately created back. Thus, the net result upon firing is the production of one token in the `GFP_mrna` place and the expenditure of one token in `3OC6HSL_LuxR_complex` place.

On the contrary, inhibitory regulation is modeled, at the PNs level, with an additional transition competing for the tokens in input to the main transition.

2.3 Compiled signaling constructs

The PNs structures produced upon compilation of signaling constructs are transitions and arcs that link places in the top-level net (i.e., BiSDL `SCOPE`s).

For example, Listing 17 compiles into the NWNs model in Figure 10. In Figure 10.a, `p1_net` is a net token in place `s1`, and `p2_net` is a net token in place `s2`. Their internal machinery is represented in Figure 10.b and Figure 10.c, respectively. `p1_net` and `p2_net` evolution takes place entirely in their respective enclosing places: net tokens do not move across top-level places. Figure 10.b shows the PNs `p1_net` corresponding to process p1 (lines 4-6, Listing 17). This is the internal machinery of the colored (net) token inside `place s1` in Figure 10.a. The `process_1` transition gets black tokens from the input place `A_molecule` and produces black tokens in the output place

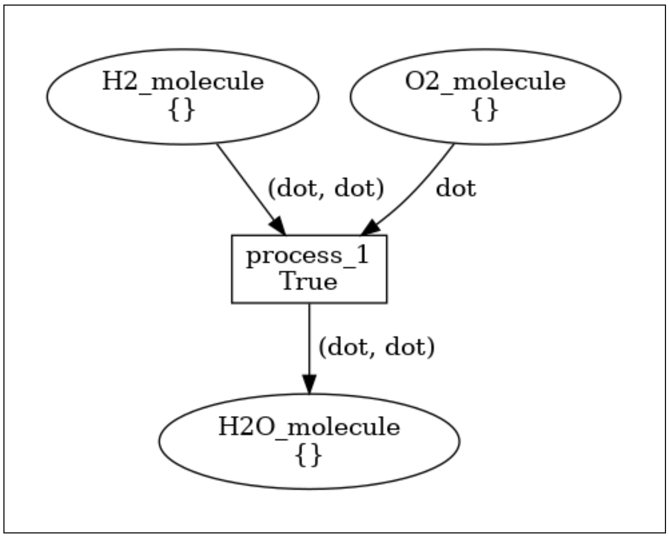

Fig. 7: PNs for the custom process corresponding to the construct for `CUSTOM PROCESS` in Section Listing 12. When the transition starts, two black tokens are consumed from the `H2_molecule` input places, one from `O2_molecule` input places, and two black tokens are produced in the output place (`H2O_molecule`).

`B_molecule`. Finally, Figure 10.c shows the PNs `p2_net`, corresponding to process p2 (lines 9-11, Listing 17), and to the internal machinery of the colored (net) token inside `place s2` in Figure 10.a. The `B_molecule_degradation` transition depletes black tokens from the input place `B_molecule`. Black tokens produced and consumed in `p1_net` places (Figure 10.b) correspond to colored tokens (strings) in the top level `s1` place (Figure 10.a). In general, a low-level black token corresponds to a top-level colored token by the same name of the place it is produced or consumed in. The transition `juxtacrine_signaling_A_molecule_s1_s2_1` is enabled by `A_molecule` colored tokens from `s1`, and outputs the same colored tokens in the output place `s2`. Note that the substitution rule `"protein", "receptor_active_protein"` has no effect in this example, thus letting `A_molecule` tokens move to the destination scope unchanged. In fact, the biological process we are modeling lets the scopes communicate through a junction (the *Channel* in Figure 1). The scope of this substitution is to model the activation of a receptor when the `JUXTACRINE_SIGNAL` construct is used to model the juxtacrine interaction between a ligand and a membrane receptor, as illustrated in the next example.

The PNs structure built after a `DIFFUSION` construct consists, in general, of two transitions per signal. One transition consumes tokens from the first <entity_name> and produces the same amount of tokens in the second <entity_name>. The other transition works in the opposite direction. When multipliers are specified for a `signal`, the PNs structure produced will be composed of as many pairs of transitions as specified by the multiplier.

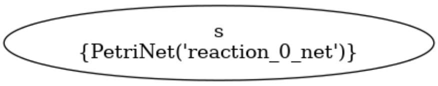

(a) Top-level PNs. `place s` represents the biological district where the reaction occurs. The reaction is secluded in the colored (net) token `PetriNet('reaction_0_net')`.

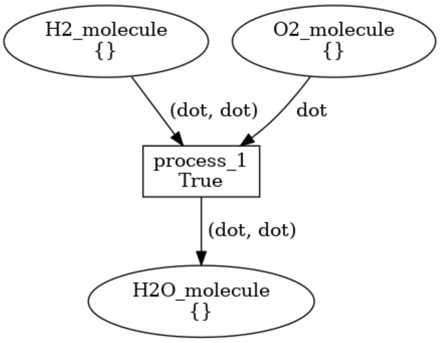

(b) Bottom-level PNs. This is the internal machinery of the colored (net) token inside `place s` (left), where the reaction at line 6, Listing 12, occurs.

Fig. 8: Graphical representation of the PNs generated by the compiled BiSDL example in Listing 12.

As an example, Listing 20 compiles into NWNs structure in Figure 12. Figure 12.a shows the top-level net structure. places `s` and `t` are mutually connected by one couple of `diffusion_A_molecule_<N>` transitions allowing `"molecule_A"` colored tokens bi-directional passage, and by two couples of `diffusion_C_molecule_<M>` transitions for `"molecule_C"` tokens passage. Such structure allows `C_molecule` to traverse the membrane with twice the probability with respect to `A_molecule`.

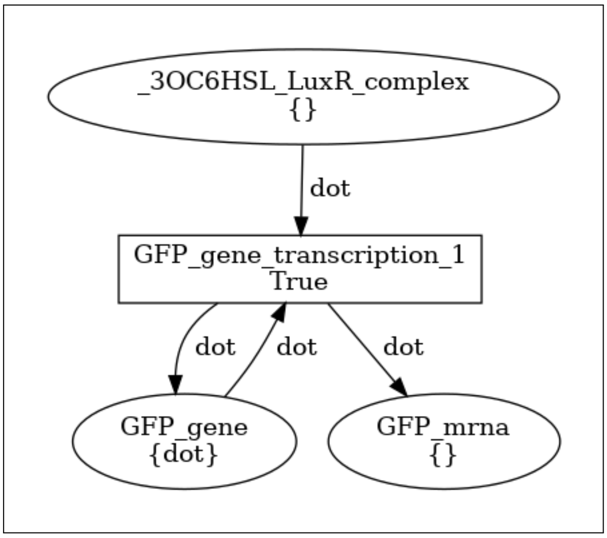

Fig. 9: PNs for the transcription process, generated from Listing 2, the BiSDL code for `TRANSCRIPTION` in Section 1.1.

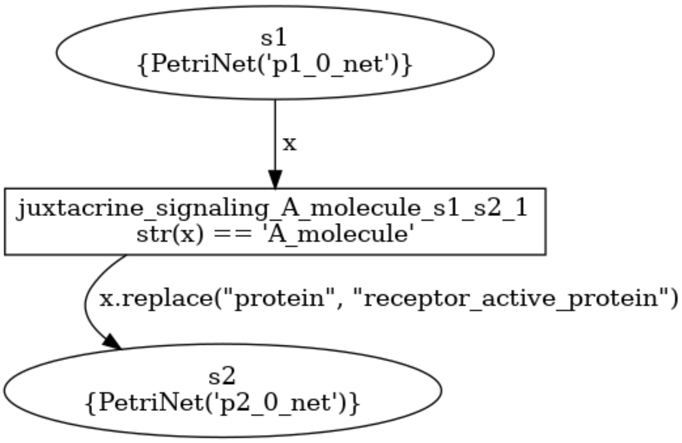

(a) Top-level PNs. `p1_net` is a net token in place s1 and `p2_net` is a net token in place s2. Their internal machinery is represented in (b) and (c), respectively.

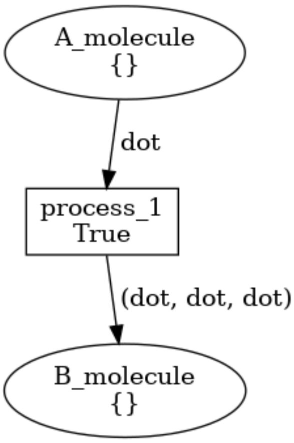

(b) Bottom-level PNs `p1_net` corresponding to the net token in place s1 (a).

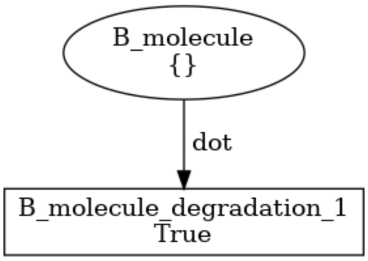

(c) Bottom-level PNs `p2_net` corresponding to the net token in place s2 (a).

Fig. 10: Graphical representation of the PNs generated by the compiled BiSDL example in Listing 17.

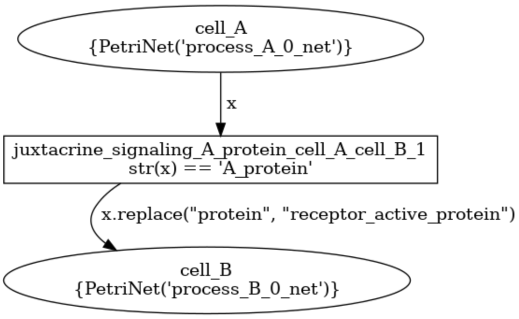

(a) Top-level PNs. `process_A_net` is a net token in place `cell_A` and `process_B_net` is a net token in place `cell_B`. Their internal machinery is represented in (b) and (c), respectively.

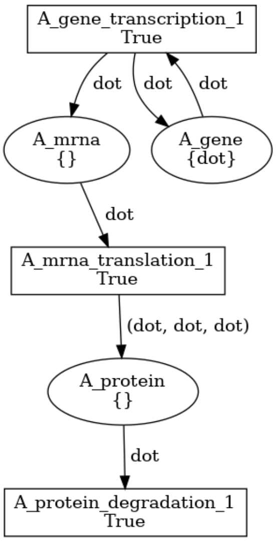

(b) Bottom-level PNs `process_A_net` corresponding to the net token in place `cell_A` (a).

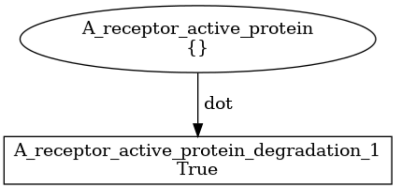

(c) Bottom-level PNs `process_B_net` corresponding to the net token in place `cell_B` (a).

Fig. 11: Graphical representation of the PNs generated by the compiled BiSDL example in Listing 18.

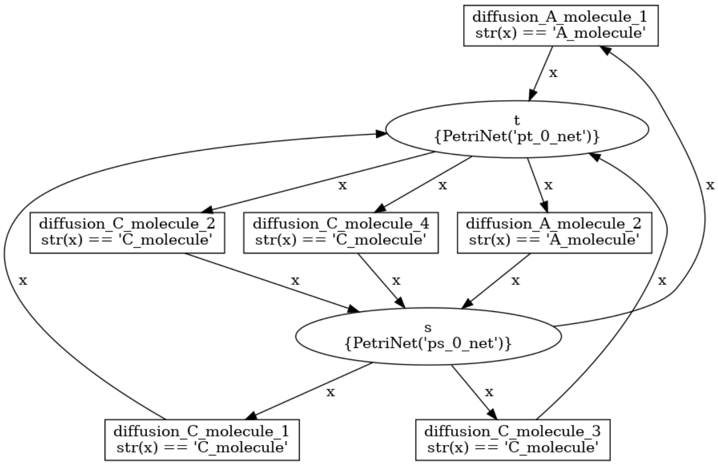

(a) Top-level PNs. `ps_0_net` is a net token in place s and `pt_0_net` is a net token in place t. Their internal machinery is represented in (b) and (c), respectively.

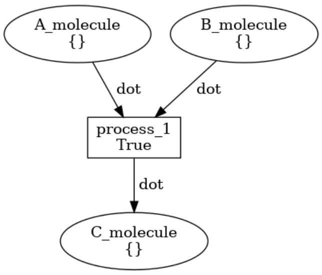

(b) Bottom-level PNs `ps_0_net` corresponding to the net token in place s (a).

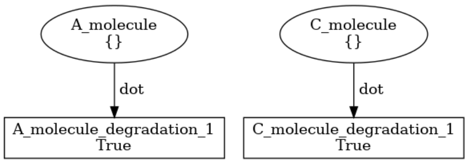

(c) Bottom-level PNs `pt_0_net` corresponding to the net token in place t (a).

Fig. 12: Graphical representation of the PNs generated by the compiled BiSDL example in Listing 20.
